# Greater pQCT Calf Muscle Density Is Associated with Lower Fracture Risk, Independent of FRAX, Falls and BMD: A Meta‐Analysis in the Osteoporotic Fractures in Men (MrOS) Study

**DOI:** 10.1002/jbm4.10696

**Published:** 2022-11-22

**Authors:** Nicholas C. Harvey, Eric Orwoll, Jane A. Cauley, Timothy Kwok, Magnus K. Karlsson, Björn E. Rosengren, Eva Ribom, Peggy M. Cawthon, Kristine Ensrud, Enwu Liu, Faidra Laskou, Kate A. Ward, Elaine M. Dennison, Cyrus Cooper, John A. Kanis, Liesbeth Vandenput, Mattias Lorentzon, Claes Ohlsson, Dan Mellström, Helena Johansson, Eugene McCloskey

**Affiliations:** ^1^ MRC Lifecourse Epidemiology Centre University of Southampton Southampton UK; ^2^ NIHR Southampton Biomedical Research Centre University of Southampton and University Hospital Southampton NHS Foundation Trust Southampton UK; ^3^ Division of Endocrinology, Diabetes and Clinical Nutrition, School of Medicine Oregon Health & Science University Portland OR USA; ^4^ Department of Epidemiology, Graduate School of Public Health University of Pittsburgh Pittsburgh PA USA; ^5^ Department of Medicine & Therapeutics and School of Public Health The Chinese University of Hong Kong Shatin China; ^6^ Clinical and Molecular Osteoporosis Research Unit, Department of Clinical Sciences Malmo Lund University and Department of Orthopedics, Skane University Hospital Malmo Sweden; ^7^ Department of Surgical Sciences University of Uppsala Uppsala Sweden; ^8^ Research Institute California Pacific Medical Center San Francisco CA USA; ^9^ Department of Epidemiology and Biostatistics University of California San Francisco CA USA; ^10^ Medicine and Epidemiology & Community Health University of Minnesota Minneapolis MN USA; ^11^ Center for Care Delivery and Outcomes Research Minneapolis VA Health Care System Minneapolis MN USA; ^12^ Mary MacKillop Institute for Health Research Australian Catholic University Melbourne Australia; ^13^ NIHR Oxford Biomedical Research Centre University of Oxford Oxford UK; ^14^ Centre for Metabolic Bone Diseases University of Sheffield Sheffield UK; ^15^ Sahlgrenska Osteoporosis Centre, Sahlgrenska Academy University of Gothenburg Gothenburg Sweden; ^16^ Centre for Integrated Research into Musculoskeletal Ageing (CIMA), Mellanby Centre for Musculoskeletal Research University of Sheffield Sheffield UK

**Keywords:** EPIDEMIOLOGY, FRACTURE, FRAX, OSTEOPOROSIS, PERIPHERAL QUANTITATIVE COMPUTED TOMOGRAPHY, PQCT, SARCOPENIA

## Abstract

We investigated the predictive performance of peripheral quantitative computed tomography (pQCT) measures of both calf muscle density (an established surrogate for muscle adiposity, with higher values indicating lower muscle adiposity and higher muscle quality) and size (cross‐sectional area [CSA]) for incident fracture. pQCT (Stratec XCT2000/3000) measurements at the tibia were undertaken in Osteoporotic Fractures in Men (MrOS) United States (US), Hong Kong (HK), and Swedish (SW) cohorts. Analyses were by cohort and synthesized by meta‐analysis. The predictive value for incident fracture outcomes, illustrated here for hip fracture (HF), using an extension of Poisson regression adjusted for age and follow‐up time, was expressed as hazard ratio (HR) per standard deviation (SD) increase in exposure (HR/SD). Further analyses adjusted for femoral neck (fn) bone mineral density (BMD) *T*‐score, Fracture Risk Assessment Tool (FRAX) 10‐year fracture probability (major osteoporotic fracture) and prior falls. We studied 991 (US), 1662 (HK), and 1521 (SW) men, mean ± SD age 77.0 ± 5.1, 73.9 ± 4.9, 80 ± 3.4 years, followed for a mean ± SD 7.8 ± 2.2, 8.1 ± 2.3, 5.3 ± 2.0 years, with 31, 47, and 78 incident HFs, respectively. Both greater muscle CSA and greater muscle density were associated with a lower risk of incident HF [HR/SD: 0.84; 95% confidence interval [CI], 0.72–1.0 and 0.78; 95% CI, 0.66–0.91, respectively]. The pattern of associations was not materially changed by adjustment for prior falls or FRAX probability. In contrast, after inclusion of fn BMD *T*‐score, the association for muscle CSA was no longer apparent (1.04; 95% CI, 0.88–1.24), whereas that for muscle density was not materially changed (0.69; 95% CI, 0.59–0.82). Findings were similar for osteoporotic fractures. pQCT measures of greater calf muscle density and CSA were both associated with lower incidence of fractures in older men, but only muscle density remained an independent risk factor for fracture after accounting for fn BMD. These findings demonstrate a complex interplay between measures of bone, muscle size, and quality, in determining fracture risk. © 2022 The Authors. *JBMR Plus* published by Wiley Periodicals LLC on behalf of American Society for Bone and Mineral Research.

## Introduction

In a previous meta‐analysis of the Osteoporotic Fractures in Men (MrOS) cohorts, we demonstrated that dual‐energy X‐ray absorptiometry (DXA) appendicular lean mass (ALM, either crude or divided by height squared) is predictive of incident fracture independently of past falls and Fracture Risk Assessment Tool (FRAX) probability.^(^ ^1^ ^)^ However, the relationship was markedly attenuated by the addition of femoral neck (fn) bone mineral density (BMD), and indeed increasing ALM (or ALM/height^2^) appeared to be a risk factor for hip fracture after accounting for this measure. A similar result was observed in the Health ABC study,^(^ ^2^ ^)^ and we have demonstrated comparable findings among women in the Women's Health Initiative,^(^ ^3^ ^)^ consistent with earlier studies in this population.^(^ ^4^, ^5^ ^)^ Importantly, both the measure of appendicular lean mass and BMD are derived from the same instrument, namely DXA. It is well established that soft tissue can influence the measurement of BMD, potentially through magnification artifact associated with a thicker body where BMI is higher, and through altered edge detection.^(^ ^6^ ^)^ This phenomenon has been particularly discussed in terms of adipose tissue. Moreover, muscle mass is not specifically measured by DXA. Rather, it is a measure of lean mass derived as the tissue that is not fat or bone, and so lean mass is clearly not the same as muscle mass.^(^ ^7^ ^)^ Interestingly the effect was very similar when ALM rather than ALM/height^2^ was used, and when controlled for fat mass, suggesting that it is not solely a result of size adjustment. In addition to concerns about the accuracy of DXA approximations of muscle mass, there is also potential bias in the assessment of BMD from soft tissue. Furthermore, BMD is calculated from equations incorporating soft tissue mass,^(^ ^6^ ^)^ and thus the possibility of measurement artifact must be considered.

These uncertainties do not help disentangle whether the alteration of the appendicular lean mass relationship by inclusion of fn BMD is a true biological effect (that is, that muscle size itself is not an important predictor of fractures once BMD is known) or an artifact of DXA (that is, that measurement error inherent in DXA assessment of muscle had led to obfuscation of the true association). One approach to resolve this issue is to study independent muscle measures from peripheral quantitative computed tomography (pQCT), a method to assess muscle size that does not suffer from the same measurement artifact issues as DXA. We therefore used pQCT muscle data from the MrOS cohorts to investigate the predictive value of pQCT muscle measures (calf muscle density [an established surrogate for muscle adiposity and quality^(^ ^8^ ^)^] and size [cross‐sectional area, CSA]) for incident fracture, independent of fn BMD, FRAX probability, and past falls. In further exploratory analyses we tested whether associations were attenuated by inclusion of body mass index (BMI).

## Subjects and Methods

### Participants

Details of the MrOS cohort studies have been published,^(^ ^1^, ^9^, ^10^, ^11^, ^12^ ^)^ but briefly, MrOS is a multicentre study of community‐dwelling men age 65 years or older from three international cohorts, recruited and evaluated using similar protocols. To be eligible for the study, subjects had to be able to walk without aid. In the MrOS Hong Kong Study, 2000 Chinese men, age 65–92 years, were enrolled between August 2001 and February 2003.^(^ ^13^ ^)^ All were Hong Kong residents of Asian ethnicity. Stratified sampling was adopted to ensure that 33% of subjects were included in each of the following age groups: 65–69, 70–74, and ≥75 years. Recruitment notices were placed in housing estates and community centers for the elderly. In the MrOS Sweden Study, 3014 men, age 69–81 years, were enrolled between October 2001 and December 2004.^(^ ^11^, ^14^ ^)^ The cohort comprised men from the cities of Malmo, Gothenburg, and Uppsala, identified and recruited using national population registers. More than 99% were of White ethnicity. The participation rate in the MrOs Sweden Study was 45%. In the MrOS United States Study, 5994 men, aged 65–100 years, were enrolled at six sites between March 2000 and April 2002.^(^ ^15^, ^16^ ^)^ Each US clinical site designed and customized strategies to enhance recruitment of its population. Common strategies included mailings from the Department of Motor Vehicles, voter registration, and participant databases, common senior newspaper features, and advertisement and targeted presentations. Self‐defined racial/ethnic ancestry was ascertained through questionnaires at baseline (90% White).

### Exposure variables

The international MrOS questionnaire^(^ ^15^ ^)^ was administered at baseline to collect information about current smoking, number and type of medications, fracture history, family history of hip fracture, past medical history (rheumatoid arthritis), and high consumption of alcohol (three or more glasses of alcohol‐containing drinks per day), calculated from the reported frequency and amount of alcohol use. Previous fracture at baseline was recorded as all fractures after the age of 50 years, regardless of trauma. For glucocorticoid exposure, this was documented in MrOS as use at least three times per week in the month preceding the baseline assessment. Apart from glucocorticoid use and rheumatoid arthritis, there was no information on secondary causes of osteoporosis and the “Secondary Osteoporosis” input variable for FRAX probability calculation was set to no for all men. Note that glucocorticoid use and rheumatoid arthritis are both specific FRAX input variables, and were thus entered into the FRAX model for the calculation of fracture probability. Self‐reported falls during the 12 months preceding the baseline were recorded by questionnaire (past falls).

At baseline, height (centimeters) and weight (kilograms) were measured, and BMI was calculated as kilograms per square meter. Areal bone mineral density (BMD) was measured at the femoral neck, and total fat mass from whole body scans, using Hologic QDR 4500 A or W (Hologic, Bedford, MA, USA) or Lunar Prodigy (GE Lunar Corp., Madison, WI, USA) depending on the centre, with cross calibration of instruments for BMD. A *T*‐score was calculated using National Health and Nutrition Examination Survey (NHANES) young women (white) as a reference value.^(^ ^17^, ^18^ ^)^ In the subset in which the necessary variables were available, FRAX 10‐year probability of major osteoporotic fracture (MOF) (hip, humerus, vertebral, or forearm sites) was calculated using clinical risk factors described above with and without fn BMD entered into country‐specific FRAX models.

### pQCT

As documented,^(^ ^19^ ^)^ tibial pQCT scans were performed using Stratec XCT‐2000 or XCT‐3000 scanners (Stratec Medizintechnik, Pforzheim, Germany). The only difference between the XCT‐2000 and XCT‐3000 models is the gantry size. The same acquisition protocol and analysis software was used to analyze scans. Quality control was performed on a daily basis using a hydroxyapatite European forearm phantom. A precision study demonstrated that values on the two instruments were similar and within less than 0.5% for total area and from 0.5% to 1.0% for total density.^(^ ^20^ ^)^ Trained technicians followed a standardized protocol for patient positioning and scanning. A scout view was obtained prior to the pQCT scan to define an anatomic reference line for the relative location of the subsequent scans at the radius and tibia. Tibia length was determined from the medial malleolus to the medial condyle of the tibia. Within the US cohorts, pQCT measures were undertaken in the subset of individuals assessed at the Pittsburgh and Minneapolis recruitment sites. Muscle CSA and density were assessed at the 66% (US), 33% (Hong Kong), or 38% site (Sweden). Daily phantom scans were analyzed to ensure long‐term scanner stability. One slice measured at 2.5 ± 0.3 mm was obtained. Images were acquired with isotropic pixel resolution of 500 μm by using the following acquisition parameters: CT speed of 20 mm/s, 38 kVp X‐ray beam energy, and matrix size of 256 × 256.^(^ ^21^ ^)^ pQCT images were semiautomatically segmented by a single user. Peel mode 2 and contour mode 3 on Stratec analysis software (version 5.5E) were applied to analyze pQCT images. And inner density threshold of 400 mg/cm^3^ and an outer density threshold of 130 mg/cm^3^ were used to separate the cortical from trabecular bone, and to separate soft tissue from bone, respectively. Muscle measurements were automatically derived using the Stratec software package.^(^ ^21^ ^)^

In the US and Hong Kong cohorts, the tibial pQCT measure was obtained at the same visit as the DXA measure of fn BMD together with the majority of clinical risk factors. In both cohorts information on rheumatoid arthritis and family history of fracture were obtained at the original baseline visit. In Sweden, assessments were updated at the pQCT visit in Gothenburg; for the Uppsala and Malmo cohorts baseline data on clinical risk factors and fn BMD were used with the mean time from baseline to pQCT follow‐up of 5.1 ± 0.2 years and 3.0 ± 0.1 years, respectively.

### Fracture outcomes

#### Hong Kong^(^ ^22^ ^)^

Incident fractures were captured via subject follow‐up through phone call or visit to the research center, and the electronic medical record system of all local public hospitals. All fracture sites (hip, wrist, skull/face, ribs, shoulder, arm, wrist, vertebra, tibia, fibula, foot, metatarsal toes, hand, fingers, and pelvis) were recorded. Pathological fractures were excluded. Only incident fractures reported by participants and confirmed by X‐ray or medical record review were included. Deaths were verified by death certificates.

#### Sweden^(^ ^23^ ^)^

Central registers covering all Swedish citizens were used to identify the subjects and the date of death for all subjects who died during the study. For incident fracture evaluation, the computerized X‐ray archives in Malmo, Gothenburg and Uppsala were searched for new fractures occurring after the baseline visit using the unique personal registration number allocated to every Swedish citizen. If additional fractures were reported by the study subject after the baseline visit, these were only included if confirmed by physician review of radiology reports.

#### US^(^ ^15^ ^)^

Triannual questionnaires were mailed to each participant. If a participant reported a fracture, study staff conducted a follow‐up telephone interview to determine the date the fracture had occurred, a description of how the fracture occurred, the type of trauma that resulted in the fracture, the participant's location and activities at the time of the fracture, symptoms just before or coincident with the fracture, and source of medical care for the fracture. All reported fractures were centrally verified by a physician adjudicator through medical records. Deaths were verified through centralized review of state death certificates.

### Statistical methods

Fracture outcomes comprised: any fracture, osteoporotic fracture (defined according to Kanis and colleagues^(^ ^24^ ^)^ as clinical vertebral, ribs, pelvis, humerus, clavicle, scapula, sternum, hip, other femoral fractures, tibia, fibula, distal forearm/wrist), major osteoporotic fracture (hip, clinical vertebral, proximal humerus, distal forearm/wrist), and hip fracture. An extension of Poisson regression^(^ ^25^ ^)^ was used to study the association between the future risk of fracture and pQCT measures, FRAX, prior falls, and BMD. All associations were adjusted for age and time since baseline. In contrast to logistic regression, the Poisson regression uses the length of each individual's follow‐up period and the hazard function is assumed to be exp(β_0_ + β_1_ · current time from baseline + β_2_ · current age + β_3_ · variable of interest). The observation period of each participant was divided into intervals of 1 month. One fracture per person, and time to the first fracture, were counted, and time at risk was censored at the time of first fracture, loss to follow‐up, death, or end of follow‐up. Unlike a standard Cox model, the Poisson model uses a data duplication method, accounting for the competing mortality risk for fracture risk prediction.^(^ ^26^ ^)^ We initially investigated the predictive value of the two pQCT measures adjusted only for age and follow‐up time. Subsequently, we used multivariate models to investigate the predictive value of these measures independent of FRAX, prior falls, or BMD (entered into the model as fn *T*‐score). The association between the exposure and risk of fracture is expressed as the gradient of risk (GR = hazard ratio per SD increase in the exposure), together with 95% confidence intervals (CI). Two‐sided *p* values were used for all analyses. Analyses were undertaken separately within each cohort and then the β‐coefficients from each cohort were weighted according to the variance, and merged to determine the weighted mean of the coefficient and its SD (fixed‐effects meta‐analysis, since heterogeneity was low to moderate as assessed by *I* ^2^).^(^ ^27^ ^)^ The risk ratios are then given by e^(weighted mean coefficient)^. Finally, we performed exploratory analyses to investigate whether the two pQCT measures were independently predictive of fracture, and whether associations were influenced by adjustment for BMI, given the role of a fat deposition in muscle density and biomechanical links between fat, muscle and bone.^(^ ^28^ ^)^

## Results

### Characteristics of participants

The study cohorts consisted of 10,411 men who had information on the key exposures, together with prior falls and femoral neck BMD. Since pQCT measures had been obtained at a subset of individual study sites, out of the original cohort, 4174 men had measures of pQCT including 991 (US), 1662 (Hong Kong), and 1521 (Sweden) men, mean ± SD age 77.0 ± 5.1, 73.9 ± 4.9, 79.5 ± 3.4 years, followed for a mean ± SD 7.8 ± 2.2, 8.1 ± 2.3, 5.3 ± 2.0 years, respectively, with the cohort characteristics summarized in Table 1. Previous fracture was more commonly reported in Sweden (37.1%) than in the US (29.6%) and Hong Kong (15.2%). Consistent with the known country‐specific epidemiology of fracture, the highest mean FRAX major osteoporotic fracture probability (with BMD) was observed in Sweden (11.8%), followed by US (8.2%) and Hong Kong (7.2%). The pattern for FRAX hip fracture probability across cohort was similar.

**Table 1:** Baseline Characteristics and Fracture Outcomes of Study Participants by Country

Characteristic	Hong Kong	Sweden	US
*n*	1662	1521	991
Person‐years (total)	13541.8	8057.5	7777.5
Age (years), mean (range)	73.9 (66.0–94.0)	79.5 (73.1–87.7)	77.0 (69.0–93.0)
BMI, mean ± SD	23.4 ± 3.1	26.2 ± 3.3	27.8 ± 3.7
Previous fracture (%)	15.2	37.1	29.6 (*n* = 989)
Family history hip fracture, *n* (%)	1382 (5.1)	1000 (13.5)	785 (13.4)
Smoker (%)	9.8	7.8 (*n* = 1517)	2.9
Glucocorticoids (%)	0.2	1.4 (*n* = 1518)	2.3
Rheumatoid arthritis (%)	1.0	1.7 (*n* = 1507)	5.7
Excess alcohol (%)	18.8	2.2 (*n* = 1497)	7.5 (*n* = 600)
BMD FN *T*‐score, mean ± SD	−1.41 ± 0.91	−0.92 ± 1.02	−0.53 ± 1.06
Previous fall (%)	17.3	13.2	23.2
Muscle cross‐sectional area (cm^2^), mean ± SD	35.4 ± 7.0	38.9 ± 7.4	75.5 ± 11.8
Muscle density (mg/cm^3^), mean ± SD	77.0 ± 3.6	69.6 ± 3.7	70.5 ± 4.6
FRAX MOF without BMD, mean ± SD	7.9 ± 3.4 (*n* = 1382)	15.7 ± 6.7 (*n* = 976)	10.6 ± 5.0 (*n* = 478)
FRAX hip without BMD, mean ± SD	4.3 ± 3.0 (*n* = 1382)	9.6 ± 6.4 (*n* = 976)	4.8 ± 4.2 (*n* = 478)
FRAX MOF with BMD, mean ± SD	7.2 ± 3.7 (*n* = 1382)	11.8 ± 6.7 (*n* = 976)	8.2 ± 4.3 (*n* = 478)
FRAX hip with BMD, mean ± SD	3.5 ± 3.1 (*n* = 1382)	6.2 ± 6.2 (*n* = 976)	2.8 ± 3.2 (*n* = 478)
FU (years), mean ± SD	8.1 ± 2.3	5.3 ± 2.0	7.8 ± 2.2
Any fx, *n* (%)	161 (9.7)	238 (15.6)	137 (13.8)
Osteoporotic fx, *n* (%)	125 (7.5)	206 (13.5)	103 (10.4)
MOF fx, *n* (%)	94 (5.7)	180 (11.8)	68 (6.9)
Hip fx, *n* (%)	47 (2.8)	78 (5.1)	31 (3.1)

Tibial pQCT sites: 66% (US), 33% (Hong Kong) or 38% (Sweden). FN = femoral neck; FU = follow‐up; fx = fracture; MOF = major osteoporotic fracture.

### Calf muscle CSA and incident fracture

**Fig. 1:**
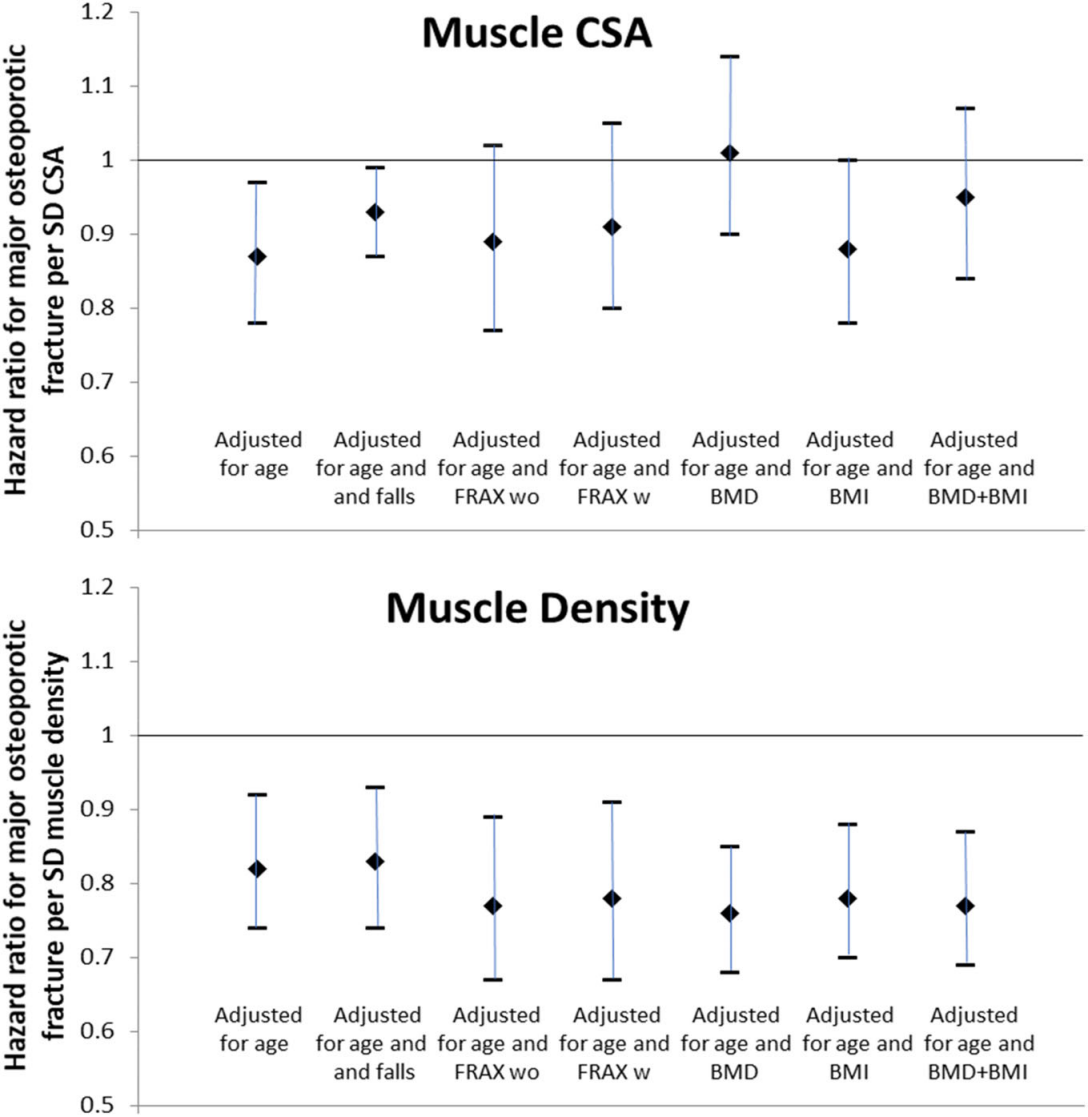
Associations between muscle CSA or muscle density and incident major osteoporotic fracture. The figures illustrate the point estimate and 95% CI around the hazard ratio per SD difference in the exposure. Models are presented adjusted for age and FU time alone and then additionally for either prior falls, FRAX MOF probability without BMD, FRAX MOF probability with BMD, femoral neck BMD *T*‐score, BMI or BMI and femoral neck BMD *T*‐score. *N* = 4174 except for +FRAX with and without BMD (*n* = 2836). CSA = cross‐sectional area; FU = follow‐up; w = with; wo = without.

Greater cross‐sectional muscle area was associated with reduced risk of all fracture outcomes (Table 2, Fig. 1). For example, the gradient of risk (GR) for hip fracture was 0.84 (95% CI, 0.72–1.0). However, for all the outcomes, additional adjustment for fn BMD *T*‐score markedly attenuated the association toward the null hypothesis of no association, with the 95% CI spanning unity in every case. (For example, hip fracture: 1.04; 95% CI, 0.88–1.24.) The separate adjustment for FRAX probability of major osteoporotic fracture with or without fn BMD was associated with a more modest attenuation in the associations. Adjustment for prior falls or BMI had little effect on the associations. Muscle CSA‐fracture associations appeared independent of muscle density. Results were consistent across the cohorts, and Table 2 summarizes these, combined through fixed effects meta‐analysis.

**Table 2:** Associations between Muscle Cross‐Sectional Area and Incident Fracture (US, Hong Kong, and Sweden)

	Adjusted for	Any fx	Ost fx	MOF	Hip fx
Muscle cross‐sectional area	Base: Age and follow‐up time	0.88 (0.80, 0.96)	0.87 (0.79, 0.97)	0.87 (0.78, 0.97)	0.84 (0.72, 1.00)
	Base + FN BMD *T*‐score	0.98 (0.90, 1.08)	0.99 (0.89, 1.10)	1.01 (0.90, 1.14)	1.04 (0.88, 1.24)
	Base + FRAX MOF wo	0.88 (0.79, 0.99)	0.85 (0.75, 0.96)	0.89 (0.77, 1.02)	0.83 (0.69, 1.01)
	Base + FRAX MOF w	0.90 (0.81, 1.01)	0.87 (0.77, 0.98)	0.91 (0.80, 1.05)	0.87 (0.71, 1.05)
	Base + prior falls	0.88 (0.80, 0.97)	0.87 (0.79, 0.96)	0.93 (0.87, 0.99)	0.85 (0.72, 1.00)
	Base + BMI	0.88 (0.79, 0.97)	0.87 (0.78, 0.98)	0.88 (0.78, 1.00)	0.88 (0.74, 1.06)
	Base + FN BMD *T*‐score and BMI	0.92 (0.84, 1.02)	0.93 (0.83, 1.03)	0.95 (0.84, 1.07)	0.97 (0.81, 1.16)
	Base + muscle density	0.87 (0.80, 0.95)	0.87 (0.78, 0.96)	0.87 (0.78, 0.97)	0.84 (0.72, 0.99)

Values are gradient of risk (GR) (95% CI). Models are presented adjusted for age and FU time alone and then additionally for either prior falls, FRAX MOF probability without BMD (FRAX MOF wo), FRAX MOF probability with BMD (FRAX MOF w), BMI, FN BMD *T* score or muscle density. *N* = 4174 except for +FRAX with and without BMD (*n* = 2836). BMI = body mass index; FN = femoral neck; FU = follow‐up; Fx = fracture; MOF = major osteoporotic fracture; Ost = osteoporotic; w = with; wo = without.

### Calf muscle density and incident fracture

Greater muscle density was associated with lower risk of incident fracture at all sites (Table 3, Fig. 1). For example, hip fracture GR: 0.78 (95% CI, 0.66–0.91). Adjustment for prior falls or BMI did not materially affect the associations. However, in contrast to the findings with muscle CSA, adjustment for fn BMD *T*‐score appeared, if anything, to strengthen the relationship (hip fracture GR: 0.69; 95% CI, 0.59–0.82). A similar effect was noted at the other fracture sites (any, osteoporotic, major osteoporotic). Consistent with this finding, adjustment for FRAX probability of major osteoporotic fracture, calculated with or without fn BMD, appeared to marginally strengthen the associations. It should be noted that although the point estimates were lower, the 95% CI still substantially overlapped between the different adjustments. Calf muscle density‐fracture associations appeared independent of muscle CSA. Again, results were consistent across the cohorts, and Table 3 summarizes these, combined through fixed effects meta‐analysis.

**Table 3:** Associations Between Muscle Density and Incident Fracture (US, Hong Kong and Sweden)

	Adjusted for	Any fx	Ost fx	MOF	Hip fx
Muscle density	Base: Age and follow‐up time	0.85 (0.78, 0.93)	0.82 (0.74, 0.90)	0.82 (0.74, 0.92)	0.78 (0.66, 0.91)
	Base + FN BMD T‐score	0.80 (0.73, 0.87)	0.76 (0.69, 0.84)	0.76 (0.68, 0.85)	0.69 (0.59, 0.82)
	Base + FRAX MOF wo	0.79 (0.71, 0.88)	0.78 (0.69, 0.88)	0.77 (0.67, 0.89)	0.73 (0.60, 0.89)
	Base + FRAX MOF w	0.81 (0.72, 0.90)	0.79 (0.70, 0.89)	0.78 (0.67, 0.91)	0.74 (0.61, 0.90)
	Base + prior falls	0.86 (0.78, 0.94)	0.82 (0.75, 0.91)	0.83 (0.74, 0.93)	0.78 (0.66, 0.92)
	Base + BMI	0.82 (0.74, 0.90)	0.78 (0.70, 0.87)	0.78 (0.70, 0.88)	0.72 (0.61, 0.86)
	Base + FN BMD T‐score and BMI	0.81 (0.74, 0.89)	0.77 (0.70, 0.86)	0.77 (0.69, 0.87)	0.71 (0.60, 0.84)
	Base + cross sectional area	0.85 (0.77, 0.93)	0.81 (0.74, 0.90)	0.82 (0.73, 0.92)	0.77 (0.65, 0.91)

Values are gradient of risk (GR) (95% CI). Models are presented adjusted for age and FU time alone and then additionally for either prior falls, FRAX MOF probability without BMD (FRAX MOF wo), FRAX MOF probability with BMD (FRAX MOF w), BMI, FN BMD *T*‐score or muscle cross‐sectional area. *N* = 4174 except for +FRAX with and without BMD (*n* = 2836). BMI = body mass index; Fx = fracture; FN = femoral neck; FU = follow‐up; MOF = major osteoporotic fracture; Ost = osteoporotic; w = with; wo = without.

## Discussion

We have demonstrated, consistent with our previous findings with DXA appendicular lean mass, that in this large population of older men across three countries, calf muscle CSA, assessed by pQCT, is only modestly predictive of incident fracture and this relationship is no longer apparent after adjustment for fn BMD. In contrast, calf muscle density, again assessed by pQCT, remained predictive of fracture outcomes after adjustment for fn BMD, and independently of adjustment for FRAX probability, prior falls, and BMI.

### Muscle CSA and density in previous pQCT fracture studies

There are very few studies in the existing literature that have prospectively examined the predictive value of pQCT muscle measures for incident fracture. These vary in site between calf and thigh, and we are not aware of any study that has directly compared the predictive capacity of these sites for incident fractures or falls. A study of 1163 men, mean age 77.2 ± 5.2 years, in the US MrOS cohort examined associations between bone‐muscle ratios (strength, mass, and area) and incident fracture.^(^ ^21^ ^)^ Lower bone to muscle ratios were associated with incident fracture, but a lower area ratio did not remain predictive after adjustment for total hip BMD, potentially consistent with our current results, and with our previous findings relating to appendicular lean mass, although use of the bone‐muscle ratio of course obscures whether the origin of an association is with the bone or the muscle component. Assessing muscle area at the mid‐thigh by axial CT in 3762 older individuals (1838 men and 1924 women; aged 66–96 years) in the AGES‐Reykjavik study, investigators observed an association between smaller muscle area and increased risk of fracture in both sexes.^(^ ^29^ ^)^ However in this study, there was no investigation of adjustment for DXA BMD. Again, consistent with our findings, analysis of 2941 women and men (including both White and Black ethnicities), age 70–79 years, in the Health ABC study, suggested that both lower thigh muscle CSA (hazard ratio/SD decrease: 1.65; 95% CI, 1.16–2.34) and lower thigh muscle density (1.58; 95% CI, 1.18–2.12) were associated with greater risk of hip fracture.^(^ ^30^ ^)^ Adjustment for total hip BMD made negligible difference to the association with thigh muscle density, but attenuated the muscle CSA‐fracture association to near unity. Our findings thus confirm, in men, this previous observation in women, extending the observations to the calf muscle site, in an independent cohort of older men, and expand the investigation to the additional relationships with FRAX probability, BMI, and prior falls.

Although the majority of sarcopenia definitions that incorporate an estimate of muscle mass using DXA appendicular lean mass (ALM), our results have implications for the use of CT muscle measures, which can be accommodated in the most recent guidelines from the European Working Group on Sarcopenia in Older people.^(^ ^31^ ^)^ Consistent with our findings in MrOS, where the predictive capacity for fracture of sarcopenia definitions incorporating DXA ALM was attenuated by adjustment for femoral neck BMD,^(^ ^32^ ^)^ the present results suggest that a similar situation would arise using pQCT muscle CSA. Overall then, there seems little evidential support for the use of DXA or pQCT muscle mass/area measures in the assessment of sarcopenia^(^ ^28^ ^)^; muscle density might offer some extra predictive value, but of course would need to be balanced against the lack of pQCT scanners in most clinical departments and the high radiation dose associated with the standard CT body scanner. An alternative strategy, particularly with the advent of machine learning image analysis, might be opportunistic use of images obtained through routine body CT scanning, as is being undertaken for detection of vertebral fractures.^(^ ^33^ ^)^ Creatine dilution has shown some promise, and data supporting its predictive value for fracture independent of BMD have recently been published.^(^ ^34^, ^35^, ^36^ ^)^

### pQCT muscle CSA, muscle density, and possible underlying mechanisms

In our previous analyses relating DXA ALM in either the MrOS cohorts^(^ ^1^, ^32^ ^)^ or the Women's Health Initiative,^(^ ^3^ ^)^ we observed that this DXA‐derived measure was modestly predictive of incident fracture, but was attenuated to the null, or even reversed, by adjustment for femoral neck BMD *T*‐score. There are of course biomechanical, hormonal, and measurement reasons for an association between DXA ALM and fn BMD^(^ ^37^, ^38^ ^)^ The resulting caveats with the DXA measure led us to hypothesize that muscle area, if measured using a different modality, might provide a better predictor of incident fracture, with more independence from fn BMD. In fact, in the present study, calf muscle CSA from pQCT behaves in a rather similar fashion to DXA ALM, in that its predictive value for fracture is removed by adjustment for fn BMD. In contrast, the association between calf muscle density and incident fracture appeared independent of this adjustment. Our findings are thus consistent with the few previous studies, albeit measuring muscle at other sites. Although muscle density is suggested to be a marker of muscle adiposity and quality, the latter remains a poorly defined concept, and the determinants of muscle density have not been well characterized.^(^ ^8^ ^)^ This measure has, however, been associated with physical performance and risk of falling.^(^ ^35^, ^39^ ^)^ It is proposed that muscle density at least partly represents adipose content of muscle tissue, but the scan resolution is of course insufficient to differentiate between intra‐myocellular and inter‐myocellular deposition.^(^ ^8^ ^)^ Correlation of muscle density with total‐body or visceral fat depots is also poorly understood, and it was notable that additional adjustment for BMI with or without fn BMD did not appear to alter the associations materially. Thus, the mechanistic underpinning of our observations awaits clarification from future studies.

### Strengths and limitations

We studied three well‐characterized cohorts drawn from general populations with standardized assessments and prospective recording of fractures. However, there are some limitations that should be considered in the interpretation of our findings.^(^ ^15^ ^)^ First, the population studied was male, and of an older age range (64–99 years), with mostly White or Asian participants, thus limiting generalizability of our findings. Second, the definition of glucocorticoid use differed from those usually specified for incorporation into FRAX. Third, there was no information on causes of secondary osteoporosis (other than rheumatoid arthritis and glucocorticoids), and this variable was therefore set to null. (Note that both glucocorticoid use and rheumatoid arthritis are entered into the FRAX model as specific input variables and therefore were considered in the analysis). The effect of these considerations on our findings is uncertain, but may have led to an underestimation of risk by FRAX. Fourth, we did not have every single FRAX input variable obtained contemporaneously with the pQCT measures. However, the time between baseline and pQCT assessment was small and findings were consistent across the cohorts. Finally, the level of pQCT assessment at the calf differed between the cohorts. Although the absolute magnitude of measures would of course differ systematically between cohorts, the analyses were conducted within each cohort separately and then synthesized through fixed effect meta‐analysis. The heterogeneity was low to moderate consistent with the similar magnitude of effect in each cohort regardless of the level of muscle assessment, so there is no reason to think that this will have materially affected our results.

## Conclusion

pQCT measures of greater calf muscle density and CSA were both associated with lower incidence of fractures in older men, but only muscle density remained an independent risk factor for fracture after accounting for fn BMD *T*‐score. These findings have implications for the assessment of sarcopenia and demonstrate a complex interplay between measures of bone, and muscle size and quality, in determining fracture risk.

## Author Contributions

**Nicholas C Harvey:** Conceptualization; investigation; methodology; project administration; supervision; writing – original draft; writing – review and editing. **Eric S. Orwoll:** Data curation; funding acquisition; methodology; project administration; writing – review and editing. **Jane A. Cauley:** Conceptualization; data curation; funding acquisition; methodology; writing – review and editing. **Timothy Kwok:** Conceptualization; data curation; funding acquisition; methodology; writing – review and editing. **Magnus K Karlsson:** Conceptualization; data curation; funding acquisition; methodology; writing – review and editing. **Bjorn Erik Rosengren:** Conceptualization; data curation; investigation; methodology; writing – review and editing. **Eva L Ribom:** Investigation; methodology; writing – review and editing. **Peggy M Cawthon:** Conceptualization; data curation; formal analysis; funding acquisition; investigation; methodology; writing – review and editing. **Kristine Ensrud:** Conceptualization; funding acquisition; investigation; methodology; writing – review and editing. **Faidra Laskou:** Resources; writing – review and editing. **Kate A Ward:** Conceptualization; resources; validation; writing – review and editing. **Elaine M Dennison:** Conceptualization; resources; writing – review and editing. **Cyrus Cooper:** Conceptualization; funding acquisition; resources; writing – review and editing. **John Kanis:** Conceptualization; funding acquisition; resources; supervision; writing – review and editing. **Liesbeth Vandenput:** Resources; validation; writing – review and editing. **Mattias Lorentzon:** Conceptualization; funding acquisition; investigation; resources; writing – original draft; writing – review and editing. **Claes Ohlsson:** Conceptualization; data curation; funding acquisition; methodology; project administration; writing – review and editing. **Dan Mellström:** Conceptualization; data curation; methodology; resources; writing – review and editing. **Helena Johansson:** Conceptualization; data curation; formal analysis; resources; writing – original draft; writing – review and editing. **Eugene McCloskey:** Conceptualization; investigation; resources; writing – original draft; writing – review and editing.

## Conflicts of Interest

All authors have no disclosures in relation to this manuscript.

## Data Availability

Data are available on approval of an appropriate application to the MrOS Publications Committee. Further information is available from https://mrosonline.ucsf.edu/.
